# A database of marine phytoplankton abundance, biomass and species composition in Australian waters

**DOI:** 10.1038/sdata.2016.43

**Published:** 2016-06-21

**Authors:** Claire H. Davies, Alex Coughlan, Gustaaf Hallegraeff, Penelope Ajani, Linda Armbrecht, Natalia Atkins, Prudence Bonham, Steve Brett, Richard Brinkman, Michele Burford, Lesley Clementson, Peter Coad, Frank Coman, Diana Davies, Jocelyn Dela-Cruz, Michelle Devlin, Steven Edgar, Ruth Eriksen, Miles Furnas, Christel Hassler, David Hill, Michael Holmes, Tim Ingleton, Ian Jameson, Sophie C. Leterme, Christian Lønborg, James McLaughlin, Felicity McEnnulty, A. David McKinnon, Margaret Miller, Shauna Murray, Sasi Nayar, Renee Patten, Tim Pritchard, Roger Proctor, Diane Purcell-Meyerink, Eric Raes, David Rissik, Jason Ruszczyk, Anita Slotwinski, Kerrie M. Swadling, Katherine Tattersall, Peter Thompson, Paul Thomson, Mark Tonks, Thomas W. Trull, Julian Uribe-Palomino, Anya M. Waite, Rouna Yauwenas, Anthony Zammit, Anthony J. Richardson

**Affiliations:** 1 CSIRO Oceans and Atmosphere, Castray Esplanade, Hobart, Tasmania 7000, Australia; 2 School of Biological Sciences, The University of Queensland, St Lucia Queensland 4072, Australia; 3 Institute for Marine and Antarctic Studies, University of Tasmania, Private Bag 129, Tasmania 7001, Australia; 4 Plant Functional Biology & Climate Change Cluster (C3),University of Technology Sydney, PO Box 123, Broadway, New South Wales 2007, Australia; 5 Biological Sciences/Marine Research Centre/Climate Futures, Macquarie University, North Ryde, New South Wales 2109, Australia; 6 eMarine Information Infrastructure, Integrated Marine Observing System University of Tasmania, Private Bag 110, Hobart, Tasmania 7001, Australia; 7 Microalgal Services, 308 Tucker Road, Ormond Victoria 3204, Australia; 8 Australian Institute of Marine Science, PMB No 3, Townsville MC, Townsville, Queensland 4810, Australia; 9 Australian Rivers Institute, Griffith University, Nathan, Queensland 4111, Australia; 10 Natural Resources, Hornsby Shire Council, 296 Peats Ferry Rd, Hornsby New South Wales 2077, Australia; 11 CSIRO Oceans and Atmosphere, EcoSciences Precinct, GPO Box 2583, Dutton Park, Queensland 4001, Australia; 12 Antarctic Climate and Ecosystems Cooperative Research Centre, Private bag 80, Hobart, Tasmania 7001,Australia; 13 Waters, Wetlands and Coasts Science Branch, New South Wales Office of Environment and Heritage, PO Box A290, Sydney South, New South Wales 1232, Australia; 14 Centre for Tropical Water and Aquatic Ecosystem Research, ATSIP Building 145 James Cook University, Townsville, Queensland 4811, Australia; 15 Institute F.-A. Forel, Earth and Environmental Sciences, University of Geneva, 66 Bvd Carl-Vogt, 1211 Geneva 4, Switzerland; 16 Environmental Monitoring and Assessment Sciences, Department of Science, Information Technology and Innovation, EcoSciences Precinct, GPO Box 5078, Brisbane, Queensland 4001, Australia; 17 School of Biological Sciences, Flinders University, GPO BOX 2100, Adelaide, South Australia 5001, Australia; 18 CSIRO Oceans and Atmosphere, Underwood Avenue, Floreat, Western Australia 6014, Australia; 19 South Australian Research and Development Institute—Aquatic Sciences, PO Box120, Henley Beach, South Australia 5022, Australia; 20 Environment Protection Authority, Centre for Applied Science, Ernest Jones Drive, Macleod, Victoria 3085, Australia; 21 Australian Institute of Marine Science & North Australia Marine Research Alliance (NAMRA), Arafura Timor Research Facility, Darwin, Northern Territory 0810, Australia; 22 The Oceans Institute, University of Western Australia, M470, 35 Stirling Hwy, Crawley, 6009 Western Australia, Australia; 23 National Climate Change Adaptation Research Facility, Griffith University, Gold Coast, Queensland 4222, Australia; 24 Warringah Council, Civic Drive, 725 Pittwater Road, Dee Why, New South Wales 2099, Australia; 25 School of Civil, Environmental and Mining Engineering, The University of Western Australia, Crawley, Western Australia 6009, Australia; 26 Alfred Wegener Institute Helmholz Centre for Polar and Marine Research Am Handelshafen 12, D-27570 Bremerhaven, Germany and University of Bremen, 28359 Bremen, Germany; 27 New South Wales Government Food Authority, 6 Avenue of the Americas, Newington, New South Wales 2127, Australia; 28 Centre for Applications in Natural Resource Mathematics (CARM), School of Mathematics and Physics, The University of Queensland, St Lucia, Queensland 4072, Australia

**Keywords:** Community ecology, Biodiversity, Marine biology

## Abstract

There have been many individual phytoplankton datasets collected across Australia since the mid 1900s, but most are unavailable to the research community. We have searched archives, contacted researchers, and scanned the primary and grey literature to collate 3,621,847 records of marine phytoplankton species from Australian waters from 1844 to the present. Many of these are small datasets collected for local questions, but combined they provide over 170 years of data on phytoplankton communities in Australian waters. Units and taxonomy have been standardised, obviously erroneous data removed, and all metadata included. We have lodged this dataset with the Australian Ocean Data Network (http://portal.aodn.org.au/) allowing public access. The Australian Phytoplankton Database will be invaluable for global change studies, as it allows analysis of ecological indicators of climate change and eutrophication (e.g., changes in distribution; diatom:dinoflagellate ratios). In addition, the standardised conversion of abundance records to biomass provides modellers with quantifiable data to initialise and validate ecosystem models of lower marine trophic levels.

## Background & Summary

Phytoplankton are microalgae at the base of the food web and directly or indirectly support all marine life. As highly efficient primary producers they are critical to maintaining biodiversity and supporting fisheries throughout the ocean^1^. Due to their high turnover rates and sensitivity to changes in environmental conditions phytoplankton are useful indicators of changing oceanographic conditions, climate change, and deterioration in water quality^2,3^. Some phytoplankton produce toxins which may be accumulated by filter feeding shellfish, causing irritation, serious illness or death to animals and humans.

A litre of seawater may contain up to one million algal cells, representing at least 100–150 different species^4^. These include the larger phytoplankton, dominated by the diatoms and dinoflagellates, but also include flagellates and the coccoid picoplanktonic forms. Currently, 537 infraspecific dinoflagellate and 938 diatom taxa are known to inhabit coastal and oceanic waters around Australia^5,6^. The fractions of nanoplankton flagellates and coccoid picoplankton, although smaller in size, can account for up to 90% of the total phytoplankton chlorophyll under low biomiass scenarios in offshore waters^4^. The latter are difficult, or impossible, to identify with light microscopy. Including a reliable estimate of biomass, along with cell abundance will provide more realistic information about the phytoplankton community structure at a particular point in time.

Many researchers have phytoplankton data sitting around on paper records or in spreadsheets, some published and some unpublished, which may eventually be lost or misplaced. Consultancies often hold data archives from several research projects from which data are released only to the client with no time and incentive to publish. These data, archived thoroughly, standardised and freely available to a wider audience, are an invaluable resource for the research community. Small, individual datasets with limited stand alone impact, can collectively provide valuable additions to large scale spatial and temporal studies. Many of these data have previously been published in some form. Journal articles, theses and reports and especially older publications, rarely include the relevant dataset so the data are not available for use after publication unless the author releases the data to a publically accessible platform. Even when data are released, they are often only presence data and are not explicit as they could be.

**Figure 1:**
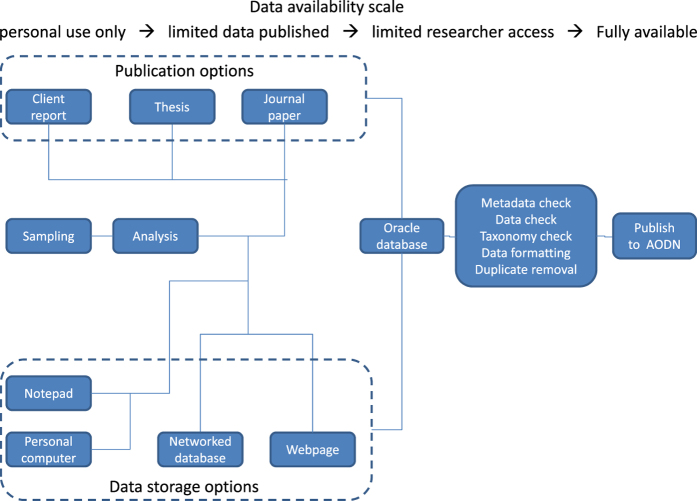
Flow diagram of data collation, verification and release to AODN.

**Figure 2:**
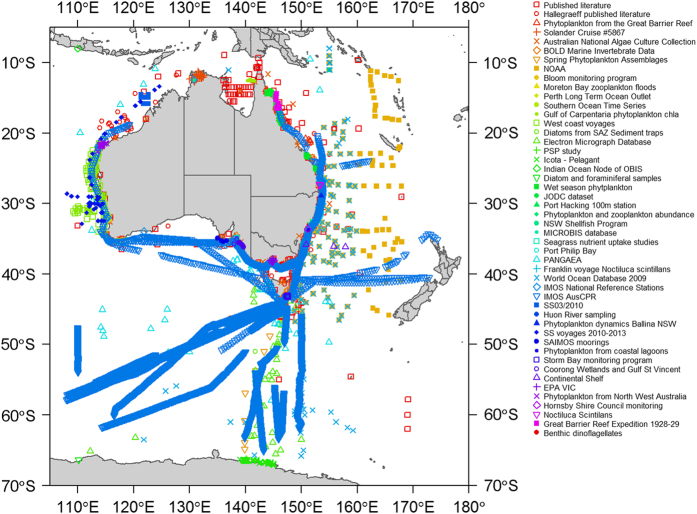
Locations of data for each project in the Australian Phytoplankton Database.

The Australian Phytoplankton Database has been collated from literature, active and retired researchers, consultancies, archives and databases (Fig. 1). Only data with the relevant corresponding metadata about collection location, date and methods has been included. Figure 2 shows the spatial extent of the records collected in this data set. Taxonomic identification of many phytoplankton taxa is fraught with difficulties, especially when limited to light microscopy. Whilst all have been standardised to the correct current classification as given by the World Register of Marine Species (WoRMS) (http://www.marinespecies.org/aphia.php?p=webservice), it is clearly not possible to verify every identification made by each analyst.

The Australian Phytoplankton Database contains data on marine phytoplankton abundance, biomass and species composition. It can be used to:

- develop distribution or biogeographic maps^4,7^;
- determine community structures and range changes over time^8,9^ or oceanographic conditions^10^;
- understand dynamics of harmful algal species to help inform the aquaculture industry^11^;
- develop inputs to climate, ecosystem and fisheries models to inform management about resources^12^.

The Australian Phytoplankton Database is available through the Australian Ocean Data Network (AODN: http://portal.aodn.org.au) portal. This portal is the main repository for marine data in Australia. The Phytoplankton Database will be maintained in the CSIRO data centre and can be updated with new records, which will automatically upload to the AODN. Researchers wishing to submit new data should contact the corresponding author or the AODN. A snapshot of the Australian Phytoplankton Database as it is at the time of this publication has been assigned a DOI and will be maintained in perpetuity by the AODN (Data Citation 1).

The Australian Phytoplankton Database has been built with ease of use and minimising user error in mind. Therefore it provides the clean data at a level that requires minimal interpretation. The CSIRO database holds all the raw data, for example original species names and ambiguous records, from these datasets, and researchers can request further information from the corresponding author if required.

## Methods

Samples were mainly collected from Niskin bottles, net drops or tows, and the Continuous Plankton Recorder (CPR). These are all standard methods of collecting phytoplankton samples^13,14^ and many are still largely reliant on a phytoplankton manual written in 1978 (ref. 15). A few samples were collected using automated samplers on moorings^16^. The sampling was done via research vessels, container ships and small boats by experienced researchers, students and volunteers. The majority of the samples were preserved using Lugol’s solution, although formalin, paraformaldehyde and glutaraldehyde have also been used. Different methods of preservation can affect the condition of the sample and which taxa are well preserved^4,17^. Samples were analysed with standard methods including light microscopy, transmission or scanning electron microscopy which are described in Hallegraeff, *et al.*^4^ All methodological variations within our phytoplankton database are detailed in the metadata where available and recorded for each data entry. Where available a citation is referenced for each project, which gives details on methodologies and limitations from that project (Table 1 (available online only)).

There were three stages in data gathering. The first stage was to conduct a literature review of Australian phytoplankton data. Any literature that contained abundance or presence data was digitised and uploaded into the CSIRO maintained Oracle database. The second stage was to scan already existing databases, such as the CSIRO data trawler, the Ocean Biogeographic Ocean System (OBIS) and the Atlas of Living Australia (AOLA). These repositories only store presence records. Relevant data were selected and uploaded into the database. The third stage was to ask researchers to contribute any other data sets that they had. All data were organised into a standard format and uploaded into the database. Data were then served to and hosted by the AODN.

All taxa have been verified as accepted species and given the currently accepted name as defined by the World Register of Marine Species database (WoRMS—http://www.marinespecies.org/aphia.php?p=webservice). If any taxa could not be verified, then a second check was done through AlgaeBase (http://www.algaebase.org/). If this did not verify that taxa as a valid name then the taxonomic level of identification was decreased to a satisfactory level or the entry removed. All abundance values were standardised to cells.l^−1^ or are given as presence only. Records of the original identifications and units were archived so any records can be checked.

Identification of the smaller phytoplankton is often to a coarser taxonomic level as many cannot be distinguished to species using light microscopy. In some studies electron microscopy has been used to determine species, but in other studies functional groups have been identified. This data set does not include accessory pigment data which can help resolve these smaller taxa^18^ although it can be thought of as a complementary dataset. Over 20 years of pigment data are available in Australia via the AEsOP database (http://aesop.csiro.au/).

Cell biovolume is calculated as per Hillebrand, *et al.*^19^ following the suggested shape factors for each genera. Size parameters were estimated from measurements taken from Australian samples or Australian references where available^4,20^ and other sources where not^21–23^. In some data sets, direct measurements of size classes (e.g., P599 the Australian Continuous Plankton Recorder Survey and P597 the IMOS National Reference Stations), were used in preference to literature values. For some taxa there was insufficient information available to estimate a biovolume, these were generally the rare taxa. Rather than estimate a size class without any information available, these have been left blank.

## Data Records

Each data record represents the abundance or presence of a phytoplankton taxa at a certain point in space and time and has been given a unique record identification number, P(project_id)_(sample_id)_(record_id). Each data record belongs to a project, with each project having a unique identification number, Pxxx. A project is defined as a set of data records which have been collected together, usually as a cruise or study with the same sampling method and having the same person counting the samples. Metadata ascribed to a project relates to all the data records within that project. Details to identify each separate project are given in Table 1 (available online only). Each sample within that project has a unique sample_id. The sample_id has not been changed from the original data set to maintain traceability. So these may be duplicated between projects but P(project_id)_(sample_id) will be a unique entity in space and time. Species abundance records within the sample are given a unique record_id.

The majority of these projects have been uploaded as part of the collation of data for this database. None have been previously published as datasets but The IMOS National Reference Stations, P599, and Continuous Plankton Recorder Survey, P597, which together constitute half the data in this database, are continually updated and available through the AODN (https://portal.aodn.org.au/search?uuid=dfef238f-db69-3868-e043-08114f8c8a94 and https://portal.aodn.org.au/search?uuid=c1344979-f701-0916-e044-00144f7bc0f4 respectively).

Table 1 (available online only) gives summary information on the project data sets, their space, time and taxonomic resolutions, numbers of samples and records available. Users can select data sets from this information and download as desired through the AODN.

## Technical Validation

The Phytoplankton Database will provide an extensive resource for phytoplankton researchers, although there are some caveats due to the variety of the sampling and analysis protocols.

The various sample collection methods infer that abundances might not always be directly comparable across projects. For example, quantitative methods such as bottle sampling (e.g., Project 599) will collect all but the rarest phytoplankton and will include the whole size spectrum, whereas semi-quantitative methods such as net sampling are selective and dependent on tow method, mesh size and the mix of species present in the water as some species may clog the net and trap smaller species that would otherwise go through the mesh (e.g., Project 509)^21^. By including all data collected using different methods and including this information as meta-data, researchers are able to analyse the relative abundance of each taxa within a project and compare across compatible projects. Metadata includes as much detail as is available about sampling methods and limitations and provides guidance to the users about the potential of each data set. Users should consider collection methods, preservation techniques and microscopic limitations when comparing datasets. All datasets have been standardised to taxa/m3 of water except P1070 where the units are taxa per gram of substrate measured. This project collected the phytoplankton by collecting substrates and analysing parts of the substrate. It is included here as the only data set on Gambierdiscus and associated benthic dinoflagellates from Australia which are important to the studies of the ciguatera.

All datasets submitted can be interpreted as confirming the presence of those species recorded. In some datasets, e.g., time series, it is possible also to infer absences, assuming that all species are looked for on each sampling occasion. Absences have been included in the data sets where the project information available allowed us the confidence to interpret such absences correctly. The interpretation of absences from other projects is at the discretion of the user. We suggest that a project-by-project approach should be taken. If a taxa is not observed at all in a project, then the absence could be due to the taxa not being present, that taxa not being of interest to the analyst, or the inability to identify that taxa. Thus, a real absence should not be inferred. If a taxa is observed in some samples of a project, it can most likely be assumed that the microscopist could identify the taxa, and that a real absence may be inferred in samples within that project where the taxa was not marked as present.

Some data records were removed when there was ambiguity as to the identification of the taxa, i.e., when the taxonomic traceability, usually from older sources, was confused or when spelling mistakes make it unclear which one of two species was meant. Species known as freshwater species were removed as the methods used to collect data were not aimed at freshwater environments and the inclusion of the odd records of these species would not be comprehensive or meaningful. Estuarine species were captured and the records kept. Data records with positions on land, with an unreal number for abundance or with impossible dates were also removed or converted to presence records.

## Usage Notes

The database contains information on the functional group of the species, which can aid analysis. Functional groups include diatoms, dinoflagellates, flagellates, ciliates (including tintinnids), silicoflagellates, and cyanobacteria. Once downloaded and binned as required, data are suitable for use in the creation of ecological indicators. For example:

- Total diatoms, dinoflagellates
- Diatom:Dinoflagellate ratio
- Total phytoplankton abundance or biomass per degree square

Abundance (cells.l^−1^) for the phytoplankton counts is given where it is available, providing more information about the productivity of an area than presence data alone. A low cell abundance may indicate a low level or production, but this may not be the case if these are large cells. The biomass data helps to show productivity of an area. The biovolume has been calculated for each cell count and when converted to biomass, is available for use by modellers and to assist in interpretation of an area’s productivity. An accepted method of converting biovolume to biomass is to assume that the cell has the density of water (1 mm^3^.l^−1^=1 mg.l^−1^)^13^. Another useful conversion is to carbon biomass; full methods are readily found in the literature^24–26^. Table 2 gives details conversions of phytoplankton size data to carbon biomass.

Some of the data records are missing dates or coordinates. It was considered useful to keep these records as the presence of a taxa in a location may still be of value. The user may estimate coordinates from the location given and would then also be aware that these would not be the exact coordinates of the sample.

**Figure 3:**
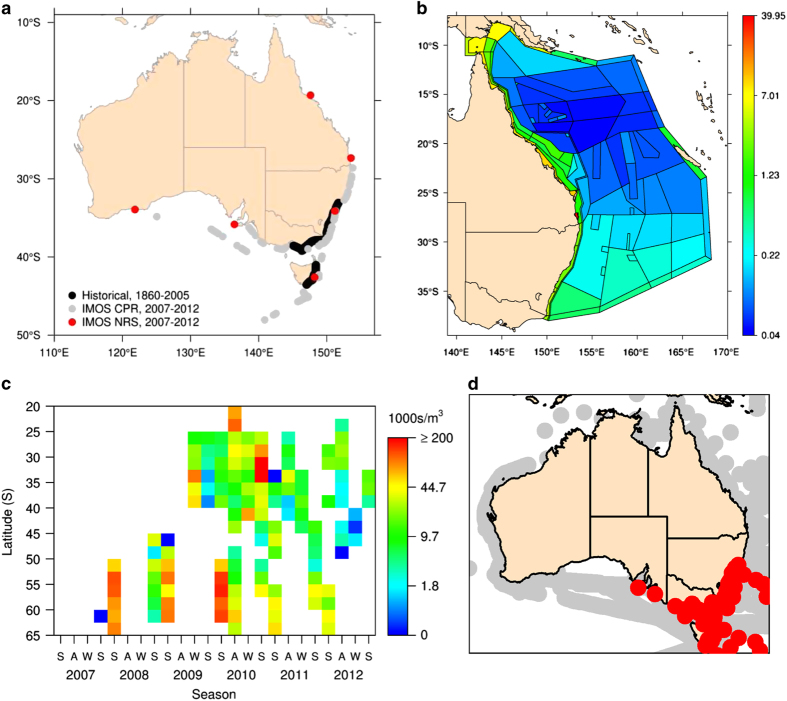
Demonstrated uses of phytoplankton data in the Australian Phytoplankton Database. (**a**) Map showing the range extension of *Noctiluca scintillans* over the past 150 years^28^; (**b**) Parameterisation of an ecosystem model (Atlantis) of diatom abundance using satellite chlorophyll Phytoplankton Functional Type analysis and the Continuous Plankton Recorder species ratios; (**c**) Diatom diversity by latitude from the Continuous Plankton Recorder; (**d**) Distribution of potentially harmful algal bloom species *Dinophysis tripos*.

Data can be analysed in many different ways and in many software applications (e.g., R, Matlab). We include here some figures created in The R Project for Statistical Computing (https://www.r-project.org) to demonstrate some potential uses of the data (Fig. 3).

In some cases, notably project 599, the IMOS National Reference Stations, the phytoplankton component of the survey is only a part of the data available. Additional biogeochemical data are available for this data set via the AODN. Some of the projects, viz. P479, P599, P597, have corresponding zooplankton data freely available in The Australian Zooplankton Database^27^. These data sets can be matched by the project_id and the sample_id which are consistent across databases. The list of citations referenced in Table 1 (available online only) will also give users information as to how this data has been previously used from the discrete projects.

## Additional information

**How to cite this article:** Davies, C. H. *et al.* A database of marine phytoplankton abundance, biomass and species composition in Australian waters. *Sci. Data* 3:160043 doi: 10.1038/sdata.2016.43 (2016).

## Supplementary Material



## Figures and Tables

**Table 1:** Summary information on the project data sets, their location, time and taxonomic resolutions, numbers of samples and records available

**Project_id**	**Project description**	**Custodian (affiliation)**	**Acknowledgement**	**Location**	**Start date**	**End date**	**No samples**	**No records**	**Taxonomic resolution**
597*	IMOS AusCPR^14^	Anthony Richardson (CSIRO)	IMOS	Australia	2007-12-17	2016-01-20	13866	1375559	Species where possible, zooplankton data available^27^
794*	NSW Shellfish Program^29,30^	Steve Brett (Microalgal Services)	Microalgal Services	Estuaries in NSW	2004-08-24	2014-08-11	11669	1340327	HAB species only
1065*	EPA VIC long term monitoring	Renee Patten (EPA VIC)	Microalgal Services	Victoria	2008-02-21	2014-07-22	715	223266	Species where possible
599*	IMOS NRS^31^	Anthony Richardson (CSIRO)	IMOS	Australia	2008-09-29	2015-12-12	490	95625	Species where possible, zooplankton and biogeochemical data available^27^
1054	Phytoplankton dynamics	David Rissik (NCCARF)		Ballina NSW	1998-11-24	2000-11-27	407	93632	Species where possible
589	Bloom monitoring program	Ian Jameson (CSIRO)		Tasmania	1992-10-12	1993-04-15	388	62076	Species of interest only
1051	Huon River sampling	Peter Thompson (CSIRO)		Huon River TAS	1996-07-02	1998-10-05	1705	44517	Species where possible; chla
796	Phytoplankton zooplankton NSW^9,10,32^	Linda Armbrecht (Macquarie University)	IMOS, OEH, NMSC	Coffs Harbours NSW	2011-05-27	2012-09-12	297	44248	Species where possible, chla
1064*	Hornsby Shire Council monitoring	Peter Coad (Hornsby Council)	Hornsby Council / Microalgal Services	Hornsby area NSW	2003-05-06	2015-06-17	149	26969	Species where possible; chla
1068	GBR expedition 1928-29	Published literature		Great Barrier Reef QLD	1928-07-25	1929-07-17	462	33675	Species where possible
1066	Phytoplankton NW Shelf	Miles Furnas (AIMS)	GBRMPA	North West Shelf WA	1997-10-26	2002-04-11	141	25803	Species where possible
807	Wet season phytoplankton	Michelle Devlin (JCU)		North Queensland	2011-02-14	2014-05-17	206	25132	Species where possible
609	Phytoplankton dynamics	Sophie C. Leterme (Flinders University)		Coorong Wetlands Gulf St Vincent SA	2010-07-01	2013-08-01	143	23171	Selected taxa identified to highest resolution possible
509	Gulf of Carpentaria phytoplankton^33–35^	Michele Burford (Griffith University)		Gulf of Carpentaria QLD	1986-05-08	1992-03-28	611	17856	Genera where possible
786	Voyage SS04/2007	Peter Thompson (CSIRO)		West Coast Australia	2007-05-16	2007-06-04	166	17264	Species where possible, chla
1056*	SAIMOS moorings	Sophie C. Leterme (Flinders University)	IMOS	South Australia	2010-07-01	2013-02-01	92	13858	Species where possible
479	Moreton Bay zooplankton floods	Julian Uribe (CSIRO)		Moreton Bay QLD	2011-01-19	2011-12-21	84	12936	Species where possible, zooplankton data available^27^
1059	Continental Shelf	Sophie C. Leterme (Flinders University)		East Coast Aus	2011-01-28	2013-07-31	208	12017	Selected taxa identified to highest resolution possible
806	Phytoplankton Great Barrier Reef	Miles Furnas (AIMS)	GBRMPA	Great Barrier Reef QLD	1986-02-07	2001-08-28	88	11748	Species where possible
790	Voyage FR10/2000	Peter Thompson (CSIRO)		West Coast Australia	2000-11-15	2000-11-26	102	10608	Species where possible
1067	NW Shelf Phyto	Paul Thomson (UWA)	IMOS	North West Shelf WA	2015-01-18	2015-01-24	46	8234	Species where possible
788	Voyage SS07/2005	Peter Thompson (CSIRO)		West Coast Australia	2005-07-21	2005-08-10	72	7488	Species where possible
784	Voyage SS08/2003	Peter Thompson (CSIRO)		West Coast Australia	2003-10-02	2003-10-21	57	5928	Species where possible
795	Perth Long Term Ocean Outlet	Peter Thompson (CSIRO)		Perth	1999-03-24	2000-03-30	64	5687	Species where possible
1057	Phytoplankton from coastal lagoons in Sydney	Shauna Murray (UTS)		Sydney NSW	2011-11-02	2014-05-02	259	5222	Species where possible
575	NOAA	NOAA		Australia	1947-03-11	1984-02-16	138	4786	Species where possible
1058	Storm Bay	Ruth Eriksen (IMAS)		Storm Bay TAS	2009-11-09	2015-04-22	85	4703	Species where possible; chla
801	PSP study	Tim Ingleton (EnvNSW)		Morpeth NSW	2001-09-03	2001-11-12	87	3306	Species where possible, chla
780	World Ocean Database 2009^36^	OBIS World Ocean Database 2009		Australia	1946-12-30	1984-02-16	118	2961	Species where possible
782	Phytoplankton Port Hacking 100 m station^37,38^	Penelope Ajani (Macquarie University)		Port Hacking NSW	1997-04-03	2009-12-07	113	2944	Species where possible, chla
559	Wood (1954).^39^	Published literature		Australia	1902-01-01	1955-01-01	33	2924	Species where possible
511	Crosby & Wood (1958).^40^	Published literature		Australia	1938-11-01	1958-01-01	32	2670	Species where possible
804	Solander cruise #5867	Diane Purcell-Meyerink (NAMRA & AIMS)	NAMRA / AIMS	Darwin & Van Diemen Gulf, NT	2013-09-09	2013-09-22	34	2530	Species where possible, chla
805*	Southern Ocean Time Series	Ruth Eriksen (CSIRO)		Southern Ocean Time Series mooring	2010-09-12	2011-04-07	24	2409	Species where possible
792	Voyage FRxx/2005	Peter Thompson (CSIRO)		West Coast Australia	1995-06-13	1995-06-14	23	2392	Species where possible
1070	Benthic dinoflagellates^41^	Michael Holmes (DSITI)		East Coast Queensland	1982-12-02	1990-02-28	889	2186	Selected genera only, units are no per g substrate
1053	SS voyages 2010-2013^42,43^	Anya Waite (UWA/AWI)		West Coast Australia	2010-07-10	2013-07-21	255	2111	Genera where possible; chla
752	Icota—Pelagant	Icota—Pelagant Icota—Pelagant		Australia	2004-01-19	2004-01-28	54	1749	Species where possible
1069	Voyage FR05/1995^44^	Peter Thompson (CSIRO)		West Coast Australia	1995-06-04	1995-06-15	24	1562	Species where possible
800	Franklin voyage CTD Noctiluca scintillans^45^	Jocelyn Dela-Cruz (UNSW)		NSW	1998-11-14	1999-02-01	1046	1046	Noctiluca scintillans only
571	CSIRO (1965).	Published literature		Port Hacking NSW	1965-04-14	1965-12-20	1	925	Species where possible
555	Rothlisberg & Pollard, *et al.* (1994).^46^	Peter Rothlisberg (CSIRO)		Gulf of Carpentaria	1988-01-01	1988-01-01	4	744	Genera where possible
778	Spring Phytoplankton Assemblages^36^	OBIS CLIVAR-SR3		Southern Ocean	2001-11-03	2001-12-10	751	751	Species where possible
799	Franklin voyage underway Noctiluca scintillans^45^	Jocelyn Dela-Cruz (UNSW)		NSW	1998-11-14	1999-01-30	504	504	Noctiluca scintillans only
573	Jeffrey & Carpenter (1974).^47^	Published literature		Port Hacking NSW	1971-09-01	1972-05-16	423	423	Species where possible
567	ANZ-DiatomsII	Published literature		Australia and New Zealand	1844-01-01	1959-01-01	21	366	Species where possible
553	Revelante *et al.* (1982).^48^	Published literature		Australia			1	343	Species where possible
746	Diatoms from SAZ Sediment traps	OBIS Diatoms from SAZ Sediment traps		Australia	1997-09-04	1997-09-20	3	304	Species where possible
1050	SS03/2010	Peter Thompson (CSIRO)		NW shelf WA	2010-04-16	2010-04-25	12	304	Species where possible
529	Hallegraeff & Jeffrey (1984).^49^	Gustaaf Hallegraeff (UTAS)		Australia	1980-06-08	1982-05-19	1	299	Species of interest only
803	Port Philip Bay^50–52^	Sasi Nayar (SARDI)		Port Philip VIC	2012-12-11	2012-12-13	9	297	Genera where possible
587	Hallegraeff’s notebook	Gustaaf Hallegraeff (UTAS)		Australia	1982-07-05	1984-09-13	22	291	Species of interest only
537	Australian National Algae Culture Collection	Ian Jameson (CSIRO)		Australia	1962-01-01	2008-01-01	24	288	Species of interest only
772	Ocean Drilling Program	PANGAEA		Australia			11	264	Selected species only
766	International marine global change study	PANGAEA		Australia			1	222	Species where possible
563	Wood (1961).^53^	Published literature		Australia	1961-01-01	1961-01-01	14	192	Species where possible
565	Wood, Crosby & Cassie (1959).^54^	Published literature		Australia	1875-01-01	1959-01-01	14	192	Species where possible
533	Hiramatsu & De Deckker (1996).	Published literature		Southern Tasmania	1994-01-10	1994-01-10	5	185	Species where possible
768	Paleoenvironmental reconstructions	PANGAEA		Australia			33	176	Selected species only
748	Electron Micrograph Database	Electron Micrograph Database Electron Micrograph Database		Australia	1998-03-04	2005-03-03	45	165	Species where possible
541	Jeffrey & Hallegraeff (1987).	Gustaaf Hallegraeff (UTAS)		Eddy Mario NSW	1981-05-01	1981-05-01	1	152	Species where possible
770	Deep Sea Drilling Project	PANGAEA		Australia			8	134	Selected species only
543	LeRoi & Hallegraeff (2004).	Gustaaf Hallegraeff (UTAS)		Australia	1994-06-01	1995-05-30	3	87	Species where possible
523	Hallegraeff (1984).^55^	Gustaaf Hallegraeff (UTAS)		Australia	1979-04-01	1984-01-01	7	84	Species of interest only
591	Zooplankton community dynamics	Sarah Pausina (UQ)	Healthy Waterways	Moreton Bay QLD	2009-02-04	2011-09-28	82	82	Noctiluca Scintilans
527	Hallegraeff & Lucas (1988).^56^	Gustaaf Hallegraeff (UTAS)		Australia	1983-06-01	1988-01-01	6	81	Species of interest only
539	Jeffrey & Hallegraeff (1980).^57^	Gustaaf Hallegraeff (UTAS)		East Australian Current	1978-12-08	1978-12-08	1	79	Species where possible
802	Seagrass nutrient uptake studies^58^	Sasi Nayar (SARDI)		South Australia	2005-06-29	2006-02-27	9	75	Genera where possible
577	Wood (1963b).^59^	Published literature		Australia	1963-01-01	1963-01-01	7	65	Species where possible
545	LeRoi & Hallegraeff (2006).^60^	Gustaaf Hallegraeff (UTAS)		Tasmania Australia	1994-06-01	1995-05-30	3	58	Species of interest only
547	McMinn (1990).^61^	Published literature		Australia	1990-01-01	1990-01-01	9	58	Selected species only
569	CSIRO (1959).	Published literature		Port Hacking NSW	1959-02-10	1959-12-15	1	51	Species where possible
585	Hallegraeff’s Coral Sea Notebook	Gustaaf Hallegraeff (UTAS)		Coral Sea	1986-01-23	1986-02-02	49	49	Species of interest only
549	O’Connor *et al* (1996).	Published literature		Macquarie Harbour NSW	1995-09-04	1995-09-04	1	45	Species where possible
758	MICROBIS database^36^	MICROBIS database		Australia			12	44	Selected species only
525	Hallegraeff & Reid (1986).^62^	Gustaaf Hallegraeff (UTAS)		Australia	1978-03-28	1979-04-30	1	32	Species of interest only
531	Hallegraeff & Jeffrey (1993).^63^	Gustaaf Hallegraeff (UTAS)		NSW and TAS	1981-10-01	1984-09-15	5	32	Species of interest only
551	Revelante & Gilmartin (1982).^64^	Published literature		Australia			1	32	Species where possible
581	CSIRO Voyage: SP3	Published literature		Australia	1982-03-09	1982-03-17	6	29	Species where possible
521	Hallegraeff (1983).^65^	Gustaaf Hallegraeff (UTAS)		Australia	1981-10-01	1982-03-01	1	28	Species of interest only
583	CSIRO Voyage: SP7	Published literature		Australia	1982-07-05	1982-07-05	4	24	Genera where possible
744	Diatom and foraminiferal samples^36^	OBIS (IOC)		Southern Ocean	1997-02-11	1997-02-11	1	21	Species where possible
517	Grant & Kerr (1970).^66^	Published literature		Port Hacking NSW	1966-04-01	1966-04-01	1	20	Species where possible
513	Cummins *et al.* (2004).^67^	Published literature		Tuggerah Lakes NSW	1999-01-01	1999-01-01	1	19	Genera where possible
515	Gottschalk *et al.* (2007).^68^	Published literature		Queensland	2007-01-01	2007-01-01	3	19	Species where possible
742	BOLD Marine Invertebrate Data^36^	OBIS BOLD Marine Invertebrate Data		Australia			12	17	HAB species only
519	Hallegraeff (1981).^69^	Gustaaf Hallegraeff (UTAS)		Port Hacking NSW	1978-01-01	1978-01-01	15	15	Species of interest only
579	CSIRO Voyage: G01	Published literature		Australia	1960-02-02	1960-02-04	2	12	Species where possible
764	Climate	PANGAEA		Australia			9	9	One species only
754	Indian Ocean Node of OBIS	OBIS IndOBIS—Indian Ocean Node of OBIS		Indian Ocean	1903-07-02	1930-07-02	1	6	Selected species only
756	JODC dataset	JODC dataset		Australia	1977-06-28	1977-06-29	1	6	Species where possible
561	Wood (1963).^70^	Published literature		North West Australia	1963-01-01	1963-01-01	1	6	Selected species only
557	Thompson *et al.* (2008).^71^	Published literature		Huon River TAS	1997-01-01	2005-01-01	1	5	Selected species only
Organised by number of records (* ongoing datasets)									

**Table 2:** Information for converting biovolume V (μm^3^) to carbon biomass B (pgC.cell^−1^).

**Group**	**Equation**
Diatoms^24^	B=0.288×V^0.811^
Dinoflagellates^24^	B=0.76×V ^0.819^
Tintinnids^25^	B=444.5+0.053×V
Ciliates^24^	B=0.22×V ^0.939^
Other protists^24^ (excluding diatoms)	B=0.216×V ^0.939^
Silicoflagellates	as for diatoms
Phaeocystis antarctica^26^	Use B=9 pg C cell^−1^
