# Corrigendum: A database of marine phytoplankton abundance, biomass and species composition in Australian waters

**DOI:** 10.1038/sdata.2016.111

**Published:** 2016-12-06

**Authors:** Claire H. Davies, Alex Coughlan, Gustaaf Hallegraeff, Penelope Ajani, Linda Armbrecht, Natalia Atkins, Prudence Bonham, Steve Brett, Richard Brinkman, Michele Burford, Lesley Clementson, Peter Coad, Frank Coman, Diana Davies, Jocelyn Dela-Cruz, Michelle Devlin, Steven Edgar, Ruth Eriksen, Miles Furnas, Christel Hassler, David Hill, Michael Holmes, Tim Ingleton, Ian Jameson, Sophie C. Leterme, Christian Lønborg, James McLaughlin, Felicity McEnnulty, A. David McKinnon, Margaret Miller, Shauna Murray, Sasi Nayar, Renee Patten, Tim Pritchard, Roger Proctor, Diane Purcell-Meyerink, Eric Raes, David Rissik, Jason Ruszczyk, Anita Slotwinski, Kerrie M. Swadling, Katherine Tattersall, Peter Thompson, Paul Thomson, Mark Tonks, Thomas W. Trull, Julian Uribe-Palomino, Anya M. Waite, Rouna Yauwenas, Anthony Zammit, Anthony J. Richardson

**Keywords:** Community ecology, Biodiversity, Marine biology

*Scientific Data* 3:160043 doi:10.1038/sdata.2016.43 (2016); Published 21 Jun 2016; Updated 6 Dec 2016

A series of errors in our database were brought to our attention by readers, and have been corrected in an updated version of this database, which is accessible via the AODN at the following link: https://portal.aodn.org.au/search?uuid=75f4f1fc-bee3-4498-ab71-aa1ab29ab2c0

The custodian details of several datasets were incorrect. These fields in the metadata table have been updated to correctly assign P744, P746, P748, and P778 to the Australian Antarctic Division, and P752 to the Royal Belgian Institute of Natural Sciences.

Species names and functional group assignments have been changed for a small number of records to fix identified errors. *Tripos brevis* and *Tripos arietinus* were spelt incorrectly, and have been duly corrected. Pedinellaceae was wrongly assigned to dinoflagellate as a functional group, and has now been re-assigned to flagellate. The ‘Naked flagellate’ group has been renamed ‘Flagellate’ as there is some inconsistency in the use of the term ‘Naked flagellate’ and what precisely would be included. The functional group ‘Other’, has also been excluded as this contained data that was not necessarily phytoplankton but had been found in phytoplankton counts. The macroalgae *Murrayella australica*, *Cladophora* spp., *Chlorohormidium* sp., *Eudorina* spp., *Tribonema* spp., *Chlorohormidium* spp. were also removed.

In addition to these corrections, three datasets have been extended to include more recently acquired data: P 597 IMOS Australian Continuous Plankton Recorder survey (ongoing dataset, 59089 new records as of 2016-08-31); P599 IMOS National Reference Stations (ongoing dataset, 14669 new records as of 2016-08-31); and P1068 Great Barrier Reef Expedition 1928-29 (new dataset, 1340 new records). Table 1 provides a summary of the overall change in database contents.

This dataset will continue to grow and will be regularly updated with new data and any further corrections to the data. Users can email imos-plankton@csiro.au with any comments, which will be reviewed and included in future updates if applicable. The AODN portal will always direct the user to the most recent version, the original version will remain available at http://dx.doi.org/10.4225/69/56454b2ba2f79, and interim versions will be available on request.

## Figures and Tables

**Table 1:** Summary of database contents.

**Total records**	**Original**	**Version 1**
Presence/Abundance records	222617	224262
Species level	123931	125959
Genera level	78153	78921
Higher level	20533	19382
Absence records	3399230	3499959
Total records	3621847	3724221
Taxa at species level	1499	1530
Taxa at genera level	522	512
Higher level taxa	107	89

