# A database of marine phytoplankton abundance, biomass and species composition in Australian waters

**DOI:** 10.1038/sdata.2017.42

**Published:** 2017-04-11

**Authors:** Claire H. Davies, Alex Coughlan, Gustaaf Hallegraeff, Penelope Ajani, Linda Armbrecht, Natalia Atkins, Prudence Bonham, Steve Brett, Richard Brinkman, Michele Burford, Lesley Clementson, Peter Coad, Frank Coman, Diana Davies, Jocelyn Dela-Cruz, Michelle Devlin, Steven Edgar, Ruth Eriksen, Miles Furnas, Christel Hassler, David Hill, Michael Holmes, Tim Ingleton, Ian Jameson, Sophie C. Leterme, Christian Lønborg, James McLaughlin, Felicity McEnnulty, A. David McKinnon, Margaret Miller, Shauna Murray, Sasi Nayar, Renee Patten, Sarah A. Pausina, Tim Pritchard, Roger Proctor, Diane Purcell-Meyerink, Eric Raes, David Rissik, Jason Ruszczyk, Anita Slotwinski, Kerrie M. Swadling, Katherine Tattersall, Peter Thompson, Paul Thomson, Mark Tonks, Thomas W. Trull, Julian Uribe-Palomino, Anya M. Waite, Rouna Yauwenas, Anthony Zammit, Anthony J. Richardson

**Keywords:** Community ecology, Biodiversity, Marine biology

*Scientific Data* 3:160043 doi:10.1038/sdata.2016.43 (2016); Published 21 Jun 2016; Updated 11 April 2017

The authors regret that Sarah A. Pausina was omitted in error from the author list of the original version of this Data Descriptor. This omission has now been corrected in the HTML and PDF versions of this Data Descriptor, as well as the accompanying Corrigendum.

